# Associations between genetic variants of vitamin D metabolic pathway and gestational diabetes mellitus: the potential mediation role of serum 25(OH)D_3_

**DOI:** 10.1186/s12902-026-02199-w

**Published:** 2026-02-24

**Authors:** Linlin Hua, Lina Wang, Lingling Cui, Zhiqian Li, Jiajia Chen, Bing Wang, Xia Zhang, Le Ma

**Affiliations:** 1 School of Public Health, Xi’an Jiaotong University Health Science Center, Xi’an, 710061 China; 2 Department of Nutrition and Food Hygiene, College of Public Health, Zhengzhou University, Zhengzhou, 450001 China; 3 Obstetrics, The Second Affiliated Hospital of Zhengzhou University, Zhengzhou, 450014 China

**Keywords:** Gestational diabetes mellitus, 25-hydroxyvitamin D_3_, Genetic variation, Vitamin D metabolic pathway, Vitamin D receptor

## Abstract

**Background:**

Circulating vitamin D concentrations have been recognized as a risk factor for GDM. The pathway genes of vitamin D synthesis and metabolism have been shown to be strongly associated with serum vitamin D. This study aimed to evaluate the association between genetic variations and GDM risk, and to further explore the potential mediating role of serum 25(OH)D_3_ in these relationships.

**Methods:**

A nested case-control study including 131 cases of GDM and 131 controls from the Zhengzhou Birth Cohort established since 2021. We genotyped nine SNPs using the TaqMan probe method and measured serum 25(OH)D_3_ by ELISA.

**Results:**

The *VDR* rs731236-G allele was associated with reduced GDM risk: the OR (95% CI) was 0.56 (0.34, 0.93) in the multivariable adjusted model. Conversely, the *VDR* rs7975232-A allele was associated with a higher risk of GDM (OR: 1.56; 95% CI: 1.04, 2.33). Higher 25(OH)D_3_ levels were inversely associated with GDM risk (OR: 0.36; 95% CI: 0.20, 0.67; *P*_trend_ <0.01). Each 1-unit increment of log-transformed 25(OH)D_3_ level was associated with a 79% lower risk of GDM (OR 0.21, 95% CI: 0.09, 0.46). Mediation analyses indicated that 25(OH)D_3_ explained 24.7% of the protective effect of the *VDR* rs731236 variant [Indirect effect= -0.06 (-0.12, -0.01)], and 29.6% of the detrimental effect of *VDR* rs7975232 and GDM risk [indirect effect = 0.07 (0.03, 0.13)].

**Conclusions:**

The study suggested a significant association of *VDR* rs7975232 and rs731236 polymorphisms with GDM risk among the Chinese population. Higher 25(OH)D_3_ levels were associated with a decreased risk of GDM. Additionally, circulating 25(OH)D_3_ partially mediated the association between variant genotype of SNPs at *VDR*-rs731236 and *VDR*-rs7975232 and GDM risk, but the relatively wide CI prompted cautious interpretation. These findings emphasize the important role of genetic variants in *VDR* in the pathogenesis of GDM. Further studies with larger cohort sample sizes should be conducted to confirm the present findings.

**Clinical trial number:**

Not applicable.

**Supplementary Information:**

The online version contains supplementary material available at 10.1186/s12902-026-02199-w.

## Background

Gestational diabetes mellitus (GDM) is a common metabolic complication during pregnancy characterized by increased insulin resistance and pancreatic β-cell defects that lead to glucose intolerance [1]. According to the International Diabetes Federation in 2022, GDM affected 14.0% of pregnancies globally [2]. Notably, the annual incidence of GDM in China has increased noticeably from 4% in 2010 to 21% in 2020 [3]. Maternal GDM has been consistently associated with higher risk of various short- and long-term adverse consequences for both mothers and offspring, including developing type 2 diabetes [4], cardiovascular disease [5], childhood obesity [6], and metabolic disorder later in life [7]. To date, the etiology of GDM has not been fully understood but is multifactorial, involving both maternal environmental factors (e.g., maternal nutritional status and lifestyle) and genetic variants. Given that insulin therapy and oral pharmacotherapy options are subject to cost and safety issues [8, 9], innovative approaches for preventing GDM are urgently needed.

As a pivotal secosteroid hormone, vitamin D is involved in various physiological processes, such as bone mineralization, immune responses, and calcium homeostasis [10]. Vitamin D obtained via skin and dietary sources is converted by 25-hydroxylase (encoded by *CYP2R1*) in the liver into 25-hydroxyvitamin D_3_ [25(OH)D_3_], the major circulating biomarker of vitamin D status, and then hydroxylated by 1α-hydroxylase (encoded by *CYP27B1*) to the bioactive 1,25-dihydroxyvitamin D [1,25(OH)2D] form in the kidney [11, 12]. 24α-hydroxylase (encoded by *CYP24A1*) catabolizes both 25(OH)D and 1,25(OH)2D to their inactive form. The biological functions of vitamin D are activated through the interaction of its active metabolite with a single vitamin D receptor (encoded by *VDR*) [13]. Evidence from experimental studies has indicated that vitamin D possesses antidiabetic properties, including enhancements in insulin secretion [14], regulation of insulin gene transcription [15], and protection against oxidative stress and inflammatory response [16], all of which are linked to the mechanism of GDM [17]. Impaired circulating vitamin D concentrations have been recognized as a risk factor for GDM. Multiple prospective observational studies have been dedicated to evaluating the association of vitamin D status with GDM incidence, and some, but not all, found inverse associations between serum concentrations of 25(OH)D and GDM risk [18–23]. The association between circulating vitamin D and the risk of GDM remains inconclusive.

It has been proposed that circulating levels of vitamin D are under genetic influence [24, 25]. The pathway genes of vitamin D synthesis and metabolism have been shown to be strongly associated with serum vitamin D, indicating that genetic variation in these pathways may contribute to inter-individual differences in both vitamin D levels and susceptibility to GDM. Several studies have reported the associations between single nucleotide polymorphisms (SNPs) of the vitamin D metabolic pathway and insulin resistance, and risk of developing diabetes. For instance, a recent meta-analysis of case-control studies showed that *VDR* rs7975232 gene polymorphisms increased susceptibility to GDM in African and Caucasian populations, while *VDR* rs731236 polymorphism was not associated with GDM in Asian and Caucasian populations [26]. Another meta-analysis suggested that *VDR* rs7975232 may be associated with GDM risk in Turkey and Iran populations [27]. In contrast, a case-control study indicated that the VDR gene SNPs rs7975232 and rs731236 were neither significant associations nor gene-gene interactions with GDM in Chinese pregnant women [28]. The inconsistency in evidence may be attributed to methodological variations, including inadequate matching for important confounders, such as BMI, age, and ethnicity. While previous studies have largely focused on direct genetic associations, the potential biological pathway explaining this link remains less clear. Whether vitamin D acts as a functional mediator linking genetic predisposition to the development of GDM has not been systematically examined in a prospective setting.

Therefore, the aim of the nested case-control study was to explore associations between nine SNPs on four VD metabolic pathway genes (*VDR*, *CYP2R1*, *CYP24A1*, and *CYP27B1*) and GDM risk, and to evaluate the association of serum 25(OH)D_3_ concentrations with GDM risk. Furthermore, we explored the mediation effects of 25(OH)D_3_ on associations between vitamin D metabolic pathway gene polymorphisms and the risk of GDM.

## Materials and methods

### Study design and participants

The study population was drawn from the Zhengzhou Birth Cohort (ZBC), aimed at exploring the associations of genetic variants and perinatal environmental factors with the risk of GDM. Briefly, singleton pregnant women aged 18 years or older, receiving their first prenatal visit at the study hospital before 24 gestational weeks, were continuously recruited into this cohort from 2021. Baseline information on socioeconomic status, medical history, and current pregnancy details was collected by trained staff through standardized questionnaires at enrolment (Supplementary file 1). During 24–28 weeks of gestation, fasting blood samples were acquired for DNA extraction and assessment of nutritional biomarkers, and a 75 g oral glucose tolerance test (OGTT) was administered to screen for GDM. Plasma glucose levels, including fasting plasma glucose (FPG), OGTT 1-h plasma glucose (1-h PG), and OGTT 2-h plasma glucose (2-h PG), were obtained from electronic medical records.

In the present study, the nested case-control study was performed to explore the associations between vitamin D metabolic pathway gene and serum 25(OH)D_3_ levels and GDM risk. We started with 1529 pregnant women enrolled in 2021 and excluded those with GDM in previous pregnancies, preexisting type 1 or type 2 diabetes, or other chronic diseases (e.g., hypertension, cardiovascular disease, and cancer) at baseline (*n* = 45), as well as those who were taking any medication that would interfere with hormonal balance (e.g., steroids, phenytoin, and thyroid hormones) (*n* = 168), leaving 1316 participants. According to the International Association of Diabetes and Pregnancy Study Groups (IADPSG) [29], GDM was diagnosed if the glucose levels met any of the following criteria: FPG ≥ 5.1 mmol/L, 1-h PG ≥ 10.0 mmol/L, or 2-h PG ≥ 8.5 mmol/L. We identified 161 GDM cases and confirmed that the remaining 1155 participants were free from GDM. Subjects with insufficient blood samples and missing data on questionnaires (*n* = 447) were excluded, resulting in target GDM cases (*n* = 131) and potential controls without GDM (*n* = 738). One control subject was matched to each GDM case based on maternal age (± 1 year) and body mass index (BMI, ± 0.5 kg/m^2^) before pregnancy. Ultimately, the analytic sample consisted of 131 pairs of incident GDM nested case-control participants. The flow diagram for subject selection is presented in Supplementary Figure S1.

The study was approved by the institutional review board of the Second Affiliated Hospital of Zhengzhou University (2021192) and complied with the Helsinki Declaration. Written informed consent was obtained from each participant prior to enrollment.

### Sample collection and SNPs selection and genotyping

Fasting blood samples were collected in vacutainer tubes containing EDTA and serum separator tubes. EDTA whole blood was then centrifuged for 15 min at 3500 rpm at 4℃ to separate plasma, buffy coat, and red blood cells. Serum separator tubes were kept at room temperature for 60 min to coagulate and were centrifuged for 10 min at 3500 rpm at 4℃ to separate serum. After centrifugation, all samples were immediately aliquoted and frozen at − 80 °C until genotyping and serum 25(OH)D_3_ measurements.

Target SNPs involved in vitamin D metabolism were selected from American National Center for Biotechnology Information (NCBI) (http://www.ncbi.nlm.nih.gov/SNP/) and the HapMap project which provided the genotype data obtained from Han Chinese living in Beijing. SNPs with a minor allele frequency (MAF) less than 0.05 were also excluded from the study. Finally, nine SNPs were selected, including five SNPs located on *VDR* (rs731236, rs7975232, rs739837, rs1544410, and rs2228570), two SNPs located on *CYP2R1* (rs12794714 and rs10741657), one SNP located on *CYP24A1* (rs2248359), and one SNP located on *CYP27B1* (rs10877012). Detailed information on these 9 loci is shown in Supplementary Table S1**.** The linkage disequilibrium (LD) among these SNPs was evaluated using the web-based application LDlink (https://ldlink.nih.gov/?tab=home), with all 9 SNPs in LD (r^2^ ≤ 0.8) for further analysis (Supplementary Figure S2).

Genomic DNA was extracted from the buffy coat, using the DNA Extraction kit (Bioteke Inc., Beijing, China), following the manufacturer’s instructions. Genotyping of the polymorphic sites of interest was performed by a TaqMan-based polymerase chain reaction (PCR) using QuantStudio™ 6 and 7 Flex Real-Time PCR System Software (Applied Biosystems, Foster City, CA, USA). PCR amplifications were conducted in a 10 µL volume containing 1 µL of purified DNA, 5.0 µL of TaqMan Genotyping Master Mix, 0.5 µL of 20X SNP assay mixture including probes and primers, and 3.5 µL of ultrapure water. The PCRs were conducted at the following cycling conditions: 60 °C for 30 s (Pre-PCR read), then 95 °C for 5 min, 40 cycles (5 s at 95 °C and 30 s at 60 °C), and 60 °C for 30 s (Post-PCR). Genotyping was successful in the 262 recruited participants. No deviation from the Hardy-Weinberg equilibrium (HWE) was observed.

### Measurement of serum 25(OH)D_3_ level

The concentrations of 25(OH)D_3_ were determined during the second trimester, a period characterized by substantial increased in pregnancy-related insulin resistance and pancreatic β-cell defects [30]. The serum 25(OH)D_3_ level was measured using commercial enzyme-linked immunosorbent assay (ELISA) kits (Elabcience Inc., Wuhan, China) following the manufacturer’s guidelines. Briefly, serum samples were diluted with the wash solution at 1:200 and assessed in duplicate. 50 µL of diluted sera was mixed with 50 µL of biotinylated antibodies and was incubated on the prepared ELISA plates for 45 min at room temperature. Any unbound antibody was removed by washing three times with 350 µL of wash solution, and plates were incubated with 100 µL of an enzyme conjugate for 30 min. Following a second wash, 90 µL of TMB One-Step substrate was added for 30 min. Optical density (OD) was measured at 450 nm using a microplate plate reader within 15 min of adding 50 µL of stop solution. 25(OH)D_3_ concentrations were determined in ng/mL based on 25(OH)D_3_ standard curves and corrected for the dilution factor. The linearity of the ELISA was established by injection of the reference standard in the range of 3.13–200 ng/mL (r^2^ ≥ 0.995). The limit of detection of the method for 25(OH)D_3_ was 1.88 ng/mL with the recoveries of 80.0-120.0% and the intra-assay and inter-assay coefficients variance lower than 10.0%.

### Assessment of covariates

A structured questionnaire was performed to collect baseline information on demographic characteristics and clinical risk factors by trained interviewers, including maternal age, height, weight, education status, parity, history of previous poor pregnancy outcome, and family history of chronic disease (e.g., diabetes, hypertension, cardiovascular disease, and cancer). Education status was classified into 2 levels: senior high school or lower, and college degree or higher. Prepregnancy BMI (kg/m^2^) was calculated as self-reported weight in kilograms divided by the square of height in meters. Blood pressure for each participant was the average of three measurements following standardized procedures.

### Statistical analysis

Baseline characteristics of participants were displayed as mean ± standard deviation (SD) or medians (interquartile ranges) for continuous variables and frequencies (proportions) for categorical variables. The differences in characteristics among GDM cases and controls were compared using t-tests or Wilcoxon rank-sum tests for continuous variables and the Chi-square test for categorical variables. Because of the skewed distribution of 25(OH)D_3_ concentrations, a natural logarithm transformation was applied before analyses. To assess the difference between expected and observed frequencies of the genotypes, HWE was tested using the goodness-of-fit Chi-square test (Supplementary Table S1).

Conditional logistic regression models were applied to estimate the odds ratios (ORs) and 95% confidence intervals (95% CIs) for the association between vitamin D metabolic pathway gene as well as serum 25(OH)D_3_ levels and risk of GDM. Linear regression models were used to estimate β coefficients ± standard errors for 25(OH)D_3_ levels associated with gene polymorphisms. Log-transformed 25(OH)D_3_ levels were categorized into quartiles (Q1 to Q4), with the lowest quartile as the reference group. In multivariable models, in addition to maternal age and pre-pregnancy BMI, history of previous poor pregnancy outcome (yes/no) were adjusted. Tests for trends were assessed by assigning the median value of each quartile of 25(OH)D_3_ as a continuous variable. Moreover, restricted cubic splines (RCS) with three knots were used to examine the dose-response relationships between serum levels of 25(OH)D_3_ and GDM risk. To explore the extent to which 25(OH)D_3_ (the continuous mediator variable) mediates the associations between SNPs of vitamin D metabolic pathway (exposure factor, Dominant Model of rs731236 and Recessive Model of rs7975232) and GDM risk (outcome factor), mediation analyses were established using the mediation R package, adjusting for the aforementioned covariates. A bootstrap resampling method was applied to estimate the 95% CIs of total, indirect, and direct effects [31]. Statistical analyses were performed using IBM SPSS Statistics version 25.0 and R version 4.2.2, and two-tailed *P* values < 0.05 were considered statistically significant. A post-hoc power calculation for endpoints was conducted using a two-sided alpha level of 0.05. The current sample size yielded > 89% power to detect significant associations between SNPs and GDM and the mediation findings.

## Results

### Characteristics of the study populations

Demographic characteristics of the case-control population are presented in Table 1. Compared with the control group, GDM cases were more likely to have a higher history of previous poor pregnancy outcomes (*P* < 0.05). Furthermore, women with GDM had higher blood glucose concentrations, but lower fasting insulin and serum 25(OH)D_3_ levels than those without GDM (all *P* < 0.05).

**Table 1:** Baseline characteristics of the study subjects

Variables	Non-GDM (*N* = 131)	GDM (*N* = 131)	t/χ^2^	*P* value
Maternal age, years	30.67 ± 3.32	30.74 ± 3.14	-0.172	0.86
Pre-pregnancy BMI, kg/m^2^	21.92 ± 2.54	22.21 ± 2.94	-0.839	0.40
Education status, n (%)			3.625	0.06
Senior high school or lower	58(44.27)	43(32.82)		
College degree or higher	73(55.73)	88(67.18)		
Parity, n (%)			0.825	0.36
0	89(67.94)	82(62.60)		
≥ 1	42(32.06)	49(37.40)		
History of previous poor pregnancy outcome, n (%)			**5.965**	**0.02**
Yes	4(3.05)	14(10.69)		
No	127(96.95)	117(89.31)		
Family history of chronic disease, n (%)			3.645	0.06
Yes	14(10.69)	25(19.08)		
No	117(89.31)	106(80.92)		
History of chronic disease, n (%)				0.17
Yes	2(1.53)	7(5.34)		
No	129(98.47)	124(94.66)		
SBP, mmHg	111.56 ± 11.05	110.79 ± 10.84	0.570	0.57
DBP, mmHg	66.24 ± 8.85	65.80 ± 7.93	0.419	0.68
FPG, mmol/L	4.49 ± 0.32	4.93 ± 0.48	**-8.637**	**< 0.01**
OGTT 1-h PG, mmol/L	7.09 ± 1.13	9.30 ± 1.78	**-12.028**	**< 0.01**
OGTT 2-h PG, mmol/L	6.59 ± 0.97	8.54 ± 1.32	**-13.598**	**< 0.01**
Fasting insulin, µIU/mL	20.64 ± 9.04	18.62 ± 7.54	**1.964**	**0.05**
HOMA-IR	4.10 ± 1.76	4.10 ± 1.80	0.042	0.97
25(OH)D_3_, ng/mL	18.95 (12.50)	14.44 (6.45)	**-5.628**	**< 0.01**

Abbreviations: BMI, body mass index; DBP, diastolic blood pressure; FPG, fasting plasma glucose; GDM, gestational diabetes mellitus; HOMA-IR, homeostasis model assessment of insulin resistance; OGTT, oral glucose tolerance test; SBP, systolic blood pressure; 1-h PG, 1-hour plasma glucose; 2-h PG, 2-hour plasma glucose; 25(OH)D_3_, 25-hydroxyvitamin D_3_. Bolded values indicate statistically significant (*P* < 0.05)

### Associations between vitamin D metabolic pathway gene polymorphisms and GDM risk

Associations of the nine SNPs on the *VDR*, *CYP2R1*, *CYP24A1*, and *CYP27B1* genes with the GDM risk are shown in Table 2. The *VDR* rs731236 (G) allele was inversely associated with GDM risk (OR: 0.58; 95% CI: 0.35, 0.97). After adjustment for sociodemographic factors, and family and maternal disease history, such association was slightly strengthened: pregnant women with *VDR* rs731236 (A/G or G/G) allele had a 44% decreased risk of GDM (OR: 0.56; 95% CI: 0.34, 0.93) compared with those with the A/A genotype. Conversely, mutations on *VDR* rs7975232 were associated with a higher risk of GDM; the fully adjusted OR (95% CI) was 1.56 (1.04, 2.33), comparing participants with genotype CC and AC. No significant associations were observed between the mutations of the remaining SNPs and GDM risk.

**Table 2:** Allele distribution and associations between genotype of SNPs and GDM risk

Gene	SNP	Genotype	GDM Cases n(%)	Controls n(%)	Model 1		Model 2	
					*OR* (95% CI)	*P*	*OR* (95% CI)	*P*
*VDR*	**rs731236**	AA	114(87.02)	93(70.99)	1	-	1	-
		AG	16(12.21)	36(27.48)	**0.56 (0.33**,** 0.95)**	**0.03**	**0.54 (0.32**,** 0.92)**	**0.02**
		GG	1(0.77)	2(1.53)	0.62 (0.08, 4.50)	0.63	0.66 (0.09, 4.91)	0.68
		**Dominant Model**			**0.58 (0.35**,** 0.97)**	**0.04**	**0.56 (0.34**,** 0.93)**	**0.03**
		Recessive Model			0.67 (0.09, 4.91)	0.70	0.72(0.09, 5.36)	0.75
	**rs7975232**	CC	52(39.69)	61(47.29)	1	-	1	-
		AC	47(35.88)	56(43.41)	1.00 (0.67, 1.49)	0.99	1.02 (0.68, 1.53)	0.92
		AA	32(24.42)	12(9.30)	**1.60 (1.03**,** 2.50)**	**0.04**	**1.57 (1.01**,** 2.45)**	**0.04**
		Dominant Model			1.18 (0.83, 1.68)	0.36	1.20 (0.84, 1.71)	0.32
		**Recessive Model**			**1.60 (1.07**,** 2.39)**	**0.02**	**1.56 (1.04**,** 2.33)**	**0.03**
	rs739837	GG	71(54.20)	68(51.91)	1	-	1	-
		GT	46(35.11)	52(39.69)	0.92 (0.64, 1.34)	0.68	0.94 (0.65, 1.37)	0.75
		TT	14(10.69)	11(8.40)	1.11 (0.63, 1.98)	0.72	1.08 (0.60, 1.94)	0.79
		Dominant Model			0.96 (0.68, 1.36)	0.83	0.97 (0.69, 1.38)	0.87
		Recessive Model			1.15 (0.66, 2.01)	0.62	1.10 (0.62, 1.96)	0.74
	rs1544410	CC	110(84.62)	109(83.85)	1	-	1	-
		TC	19(14.62)	20(15.38)	0.99 (0.61, 1.61)	0.96	0.92 (0.56, 1.50)	0.74
		TT	1(0.76)	1(0.77)	1.06 (0.15, 7.82)	0.95	1.09 (0.14, 8.29)	0.93
		Dominant Model			0.99 (0.61, 1.60)	0.97	0.93 (0.57, 1.50)	0.76
		Recessive Model			1.07 (0.15, 7.83)	0.95	1.10 (0.15, 8.36)	0.93
	rs2228570	GG	45(34.62)	39(30.00)	1	-	1	-
		AG	61(46.92)	68(52.31)	0.88 (0.59, 1.30)	0.51	0.86 (0.58, 1.27)	0.45
		AA	24(18.46)	23(17.69)	0.96 (0.58, 1.58)	0.88	0.97 (0.59, 1.60)	0.92
		Dominant Model			0.90 (0.62, 1.30)	0.57	0.89 (0.62, 1.29)	0.54
		Recessive Model			1.04 (0.67, 1.62)	0.87	1.06 (0.68, 1.66)	0.79
*CYP2R1*	rs10741657	GG	30(23.44)	50(39.06)	1	-	1	-
		AG	75(58.59)	51(39.84)	1.52 (1.00, 2.32)	0.05	1.46 (0.96, 2.23)	0.08
		AA	23(17.97)	27(21.10)	1.21 (0.70, 2.07)	0.50	1.20 (0.69, 2.06)	0.52
		Dominant Model			1.43 (0.96, 2.15)	0.08	1.39 (0.92, 2.08)	0.12
		Recessive Model			0.91 (0.58, 1.43)	0.69	0.93 (0.59, 1.46)	0.75
	rs12794714	GG	38(30.16)	57(45.24)	1	-	1	-
		AG	73(57.94)	48(38.10)	**1.52 (1.03**,** 2.26)**	**0.04**	1.45 (0.98, 2.16)	0.06
		AA	15(11.90)	21(16.67)	1.02 (0.56, 1.86)	0.94	1.05 (0.58, 1.93)	0.86
		Dominant Model			1.40 (0.96, 2.05)	0.08	1.36 (0.93, 2.00)	0.11
		Recessive Model			0.80 (0.46, 1.37)	0.41	0.85 (0.49, 1.46)	0.55
*CYP24A1*	rs2248359	CC	63(48.09)	46(35.11)	1	-	1	-
		CT	50(38.17)	71(54.20)	0.71 (0.49, 1.03)	0.07	0.74 (0.51, 1.08)	0.12
		TT	18(13.74)	14(10.69)	0.97 (0.57, 1.65)	0.91	1.02 (0.60, 1.74)	0.95
		Dominant Model			0.76 (0.54, 1.08)	0.13	0.79 (0.56, 1.13)	0.20
		Recessive Model			1.15 (0.69, 1.89)	0.60	1.19 (0.72, 1.97)	0.51
*CYP27B1*	rs10877012	TT	39(29.77)	47(35.88)	1	-	1	-
		GT	68(51.91)	69(52.57)	1.10 (0.74, 1.63)	0.64	1.12 (0.75, 1.66)	0.59
		GG	24(18.32)	15(11.45)	1.37 (0.82, 2.29)	0.22	1.323 (0.79, 2.21)	0.28
		Dominant Model			1.16 (0.80, 1.69)	0.44	1.162 (0.80, 1.69)	0.44
		Recessive Model			1.30 (0.83, 2.02)	0.26	1.236 (0.79, 1.93)	0.35

Abbreviations: CI, confidence interval; GDM, gestational diabetes mellitus; OR, odds ratio; SNPs, single nucleotide polymorphisms; *VDR*, vitamin D receptor. A pair of alleles such as A/G, if G is a less frequent gene, then dominant model (GA + GG vs. AA), recessive model (GG vs. GA + AA). Model 1, adjusted for age and pre-pregnancy BMI. Model 2: further adjusted for previous poor pregnancy outcome history. Bolded values indicate statistically significant (*P* < 0.05)

### Association of 25(OH)D_3_ with GDM risk

**Fig. 1:**
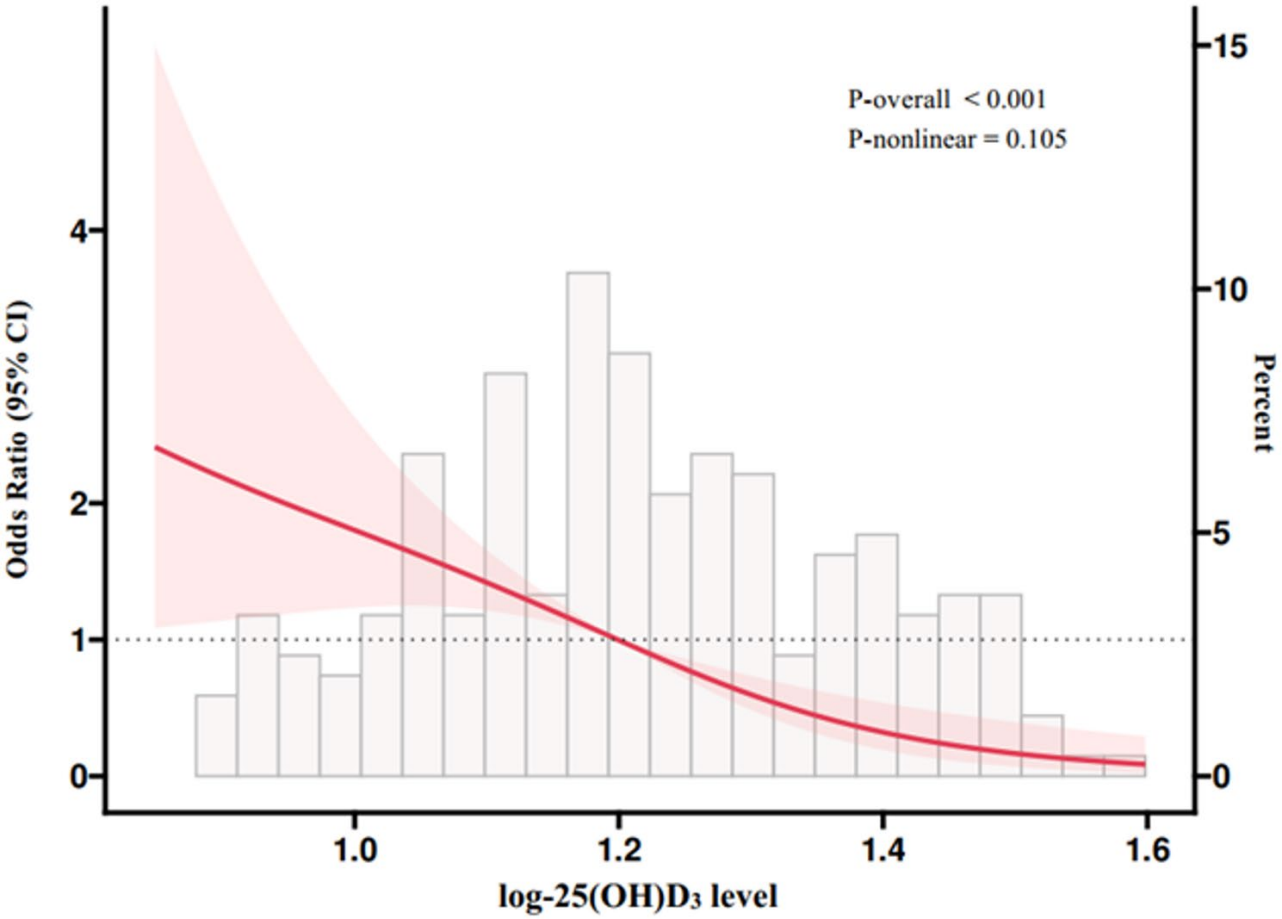
Restricted cubic spline analysis of association between log-transformed 25(OH)D_3_ levels and GDM risk. Abbreviations: BMI, body mass index; GDM, gestational diabetes mellitus; 25(OH)D_3_, 25-hydroxyvitamin D_3_. Adjusted for age and pre-pregnancy BMI, education status, history of previous poor pregnancy outcome and family history of chronic disease

Overall, mean serum 25(OH)D_3_ levels in the total population were (17.76 ± 8.71) ng/ml. In the multivariable adjusted model, the concentrations of serum 25(OH)D_3_ were inversely associated with GDM risk: the OR (95% CI) was 0.36 (0.20, 0.67; *P*_trend_ <0.01; Table 3), comparing extreme quartiles. Results from the restricted cubic spline regression showed a linear inverse association of 25(OH)D_3_ levels with GDM risk (*P*_non−linear_ = 0.11; Fig. 1). Each 1-unit increment of log-transformed 25(OH)D_3_ level was associated with a 79% lower risk of GDM (Model 2: OR 0.21, 95% CI 0.09, 0.46; Table 3).

**Table 3:** ORs (95% CIs) for GDM risk according to log-transformed 25(OH)D_3_ levels

	ORs (95% CIs) by quartiles of 25(OH)D_3_ levels				Each 1-unit log-transformed 25(OH)D_3_ increase	*P*_trend_
	Q1	Q2	Q3	Q4		
25(OH)D3 level, Median (P25, P75)	9.10 (7.54, 10.95)	14.26 (13.07, 14.93)	18.23 (16.79, 19.69)	28.32 (24.61, 31.82)		
No. Of GDM/non-GDM	41/25	48/17	28/38	14/51		
Model 1	1	1.19 (0.78, 1.81)	0.68 (0.42, 1.11)	**0.35 (0.19**,** 0.64)**	**0.20 (0.09**,** 0.43)**	**< 0.01**
Model 2	1	1.10 (0.71, 1.68)	0.66 (0.41, 1.08)	**0.36 (0.20**,** 0.67)**	**0.21 (0.09**,** 0.46)**	**< 0.01**

Abbreviations: CI, confidence interval; GDM, gestational diabetes mellitus; OR, odds ratio; 25(OH)D_3_, 25-hydroxyvitamin D_3_. Model 1, adjusted for age and pre-pregnancy BMI. Model 2: further adjusted for history of previous poor pregnancy outcome. Bolded values indicate statistically significant (*P* < 0.05)

### Associations between SNPs of vitamin D metabolic pathway and serum 25(OH)D_3_ levels

We observed significant and positive associations of two SNPs of vitamin D metabolic pathway with serum 25(OH)D_3_ concentrations (Table 4). Concretely, the *VDR* rs731236 AG and GG genotypes were associated with 3.12 ng/mL higher 25(OH)D_3_, whereas genotype AC and AA of the *VDR* rs7975232 were associated with 2.75 ng/mL lower 25(OH)D_3_ compared to noncarriers. The mutations of other SNPs were not significantly associated with the levels of 25(OH)D_3_.

**Table 4:** Association of SNPs of vitamin D metabolic pathway and serum 25(OH)D_3_ levels

Gene	SNP	Genotype	mean ± SD	Model 1		Model 2	
				β (SE)	*P* value	β (SE)	*P* value
*VDR*	**rs731236**	AA	17.22 ± 8.12	Ref	-	Ref	-
		**AG**	**20.25 ± 10.57**	**3.10 (1.35)**	**0.02**	**3.33 (1.35)**	**0.01**
		GG	11.54 ± 3.66	-6.36 (5.10)	0.21	-5.97 (5.16)	0.25
		**Dominant Model**		**2.88 (1.33)**	**0.03**	**3.12 (1.33)**	**0.02**
		Recessive Model		-6.97 (5.14)	0.18	-6.55 (5.21)	0.21
	**rs7975232**	CC	19.28 ± 9.11	Ref	-	Ref	-
		AC	17.43 ± 8.70	-1.92 (1.18)	0.11	-2.07 (1.19)	0.085
		**AA**	**14.82 ± 6.89**	**-4.60 (1.54)**	**0.01**	**-4.32 (1.55)**	**0.01**
		**Dominant Model**		**-2.71 (1.09)**	**0.01**	**-2.75 (1.09)**	**0.01**
		**Recessive Model**		**-3.67 (1.44)**	**0.01**	**-3.37 (1.45)**	**0.02**
	rs739837	GG	18.55 ± 9.09	Ref	-	Ref	-
		GT	17.27 ± 8.46	-1.31 (1.15)	0.26	-1.50 (1.16)	0.20
		TT	15.27 ± 7.01	-3.36 (1.90)	0.08	-2.86 (1.93)	0.14
		Dominant Model		-1.72 (1.08)	0.11	-1.79 (1.08)	0.10
		Recessive Model		-2.81 (1.84)	0.13	-2.31 (1.88)	0.22
	rs1544410	CC	18.03 ± 8.88	Ref	-	Ref	-
		TC	16.26 ± 7.83	-1.73 (1.53)	0.26	-1.60 (1.54)	0.30
		TT	13.40 ± 2.48	-5.16 (6.29)	0.41	-4.32 (6.37)	0.50
		Dominant Model		-1.89 (1.49)	0.21	-1.74 (1.50)	0.25
		Recessive Model		-4.87 (6.29)	0.44	-4.15 (6.37)	0.52
	rs2228570	GG	17.44 ± 8.39	Ref	-	Ref	-
		AG	18.12 ± 8.59	0.58 (1.25)	0.64	0.64 (1.25)	0.61
		AA	17.34 ± 9.79	-0.13 (1.61)	0.94	-0.26 (1.61)	0.87
		Dominant Model		0.39 (1.18)	0.74	0.39 (1.17)	0.74
		Recessive Model		-0.48 (1.41)	0.73	-0.65 (1.42)	0.65
*CYP2R1*	rs10741657	GG	18.80 ± 9.30	Ref	-	Ref	-
		AG	16.86 ± 8.56	-2.05 (1.25)	0.10	-1.87 (1.26)	0.138
		AA	18.47 ± 8.23	-0.41 (1.58)	0.80	-0.57 (1.58)	0.718
		Dominant Model		-1.59 (1.18)	0.18	-1.50 (1.18)	0.207
		Recessive Model		0.85 (1.39)	0.54	0.57 (1.39)	0.685
	rs12794714	GG	18.62 ± 9.52	Ref	-	Ref	-
		AG	17.31 ± 8.15	-1.29 (1.21)	0.29	-1.03 (1.21)	0.40
		AA	17.72 ± 8.55	-0.78 (1.70)	0.65	-0.77 (1.71)	0.65
		Dominant Model		-1.17 (1.14)	0.31	-0.96 (1.14)	0.40
		Recessive Model		-0.08 (1.57)	0.96	-0.22 (1.58)	0.89
*CYP24A1*	rs2248359	CC	17.37 ± 8.50	Ref	-	Ref	-
		CT	18.46 ± 8.94	1.06 (1.16)	0.36	0.95 (1.16)	0.42
		TT	16.44 ± 8.59	-1.10 (1.77)	0.54	-1.22 (1.78)	0.49
		Dominant Model		0.62 (1.10)	0.57	0.51 (1.11)	0.65
		Recessive Model		-1.66 (1.66)	0.32	-1.74 (1.67)	0.30
*CYP27B1*	rs10877012	TT	17.74 ± 9.18	Ref	-	Ref	-
		GT	17.79 ± 9.01	0.05 (1.21)	0.97	-0.08 (1.21)	0.95
		GG	17.70 ± 6.48	-0.10 (1.70)	0.95	0.01 (1.70)	0.99
		Dominant Model		0.02 (1.15)	0.99	-0.06 (1.15)	0.96
		Recessive Model		-0.13 (1.53)	0.93	0.06 (1.53)	0.97

Abbreviations: SD, standard deviation; SNPs, single nucleotide polymorphisms; VDR, vitamin D receptor. A pair of alleles such as A/G, if G is a less frequent gene, then dominant model (GA + GG vs. AA), recessive model (GG vs. GA + AA). Model 1: adjusted for age and pre-pregnancy BMI; Model 2: further adjusted for previous poor pregnancy outcome history. Bolded values indicate statistically significant (*P* < 0.05)

### Mediation effects of 25(OH)D_**3**_ on associations between vitamin D metabolic pathway gene polymorphisms and risk of GDM

**Fig. 2:**
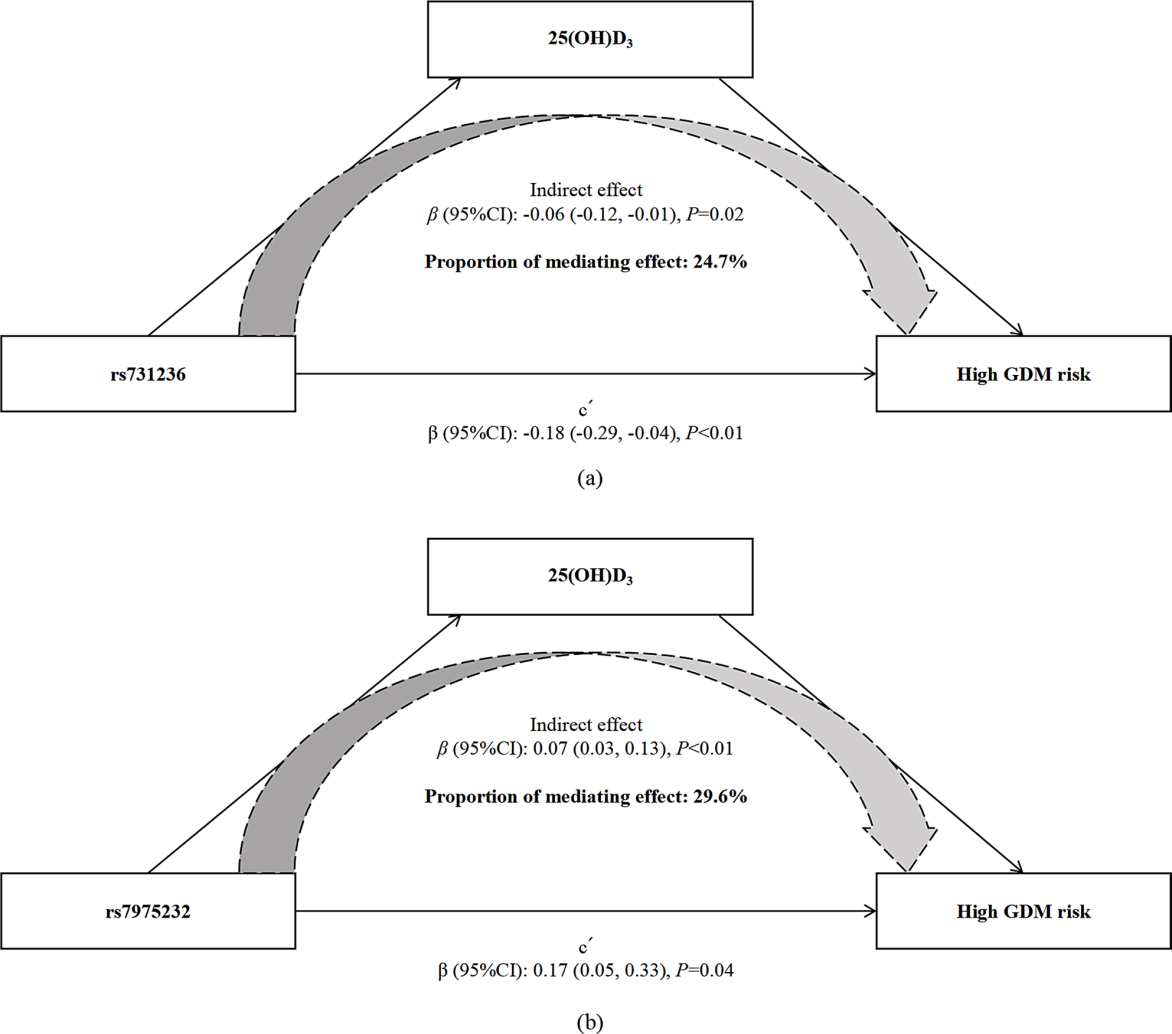
Mediation effect of 25(OH)D_3_ on the associations of SNPs with GDM. Abbreviations: BMI, body mass index; CI, confidence interval; GDM, gestational diabetes mellitus; 25(OH)D_3_, 25-hydroxyvitamin D_3_. The mediation analyses were adjusted for age, pre-pregnancy BMI, education level, previous poor pregnancy outcome history and family history of chronic disease

For two SNPs of vitamin D metabolic pathway that showed a significant association with GDM risk, we further examined the extent to which the associations of *VDR* rs731236 and rs7975232 with GDM risk were mediated by 25(OH)D_3_. Results from the mediation analysis showed that serum 25(OH)D_3_ concentrations partially mediated the relationship between *VDR* rs731236 and the risk of GDM, with a significant indirect effect (mediated effect: -0.06; 95% CI: -0.12, -0.01; *P* = 0.02; Supplementary Table S2). A significant mediation effect of 25(OH)D_3_ in the association between *VDR* rs7975232 and GDM risk was observed (mediated effect: 0.07; 95% CI: 0.03, 0.13; *P* < 0.01; Supplementary Table S2). The proportion mediated by serum 25(OH)D_3_ levels was 24.7% (95% CI: 2.8%, 76.0%; *P* = 0.02; Fig. 2A) for *VDR* rs731236, and 29.6% (95% CI: 11.4%, 70.0%; *P* < 0.01; Fig. 2B) for *VDR* rs7975232.

## Discussion

Our data suggested that *VDR* rs7975232 polymorphisms were associated with a higher risk of GDM, while *VDR* rs731236 polymorphisms were associated with a decreased risk of GDM among the Chinese population. Lower 25(OH)D_3_ levels were related to an increased risk of GDM. We further showed that vitamin D status partially mediated the associations between *VDR* rs731236 and rs7975232 and GDM risk.

Previous studies suggested that VD metabolic pathway genes were associated with the risk of GDM. As a potential candidate gene for genetic evaluations of metabolic disorders, *VDR* has been confirmed to be critically involved in the regulation of the endocrine system. A recent meta-analysis of case-control studies involving 2207 cases and 2706 controls showed that *VDR* rs7975232 gene polymorphisms increased susceptibility to GDM, particularly in the Middle Eastern population [26]. Our findings are in general consistent with a case-control study conducted in an Iranian pregnant women population, which reported a significant association between *VDR* rs7975232 (AA vs.CC, OR = 3.00; 95%CI: 1.28, 7.02) and rs731236 (TT vs.TC, OR = 0.52; 95%CI: 0.23, 0.84) polymorphisms and the initiation of GDM [32]. However, a case-control study conducted in 1684 Chinese pregnant women indicated that the VDR gene SNPs rs7975232 and rs731236 were neither significantly associated nor gene-gene interactions with GDM [28]. The conflicting results of these studies may be attributed to the differences in the genotyping methods for SNPs, ethnicity, genotype frequencies, or sample sizes. The present study showed that pregnant women with *VDR* rs731236 (A/G or G/G) allele had a 44% decreased risk of GDM, while *VDR* rs7975232-A variant was associated with a 56% increased risk of GDM. Whereas the major allele for rs7975232 differed between our study and Middle Eastern study, the high frequency of the C allele in our population was consistent with the single nucleotide polymorphism database of NCBI, indicating that the major allele of C was reasonable validity. Overall, our study supports the role of VDR gene polymorphisms in GDM susceptibility, highlighting the importance of population-specific genetic evaluation in understanding its etiology.

Evidence on the association of maternal vitamin D status with GDM risk has been mixed and controversial. In the present study, we found a significantly inverse association between 25(OH)D levels and risk of developing GDM. Our findings were largely consistent with previous investigations that emphasized the importance of assessment of vitamin D status during pregnancy [18, 20, 33]. A recent meta-analysis of 69 prospective observational studies has found that higher circulating 25(OH)D levels were associated with a 24% (RR: 0.76; 95% CI: 0.66, 0.87) reduction in the risk of GDM [33]. Similar findings were revealed in another meta-analysis, in which vitamin D deficiency was significantly associated with an increased risk of GDM (OR: 1.26; 95% CI: 1.13, 1.41) [20]. Although most included studies in this meta-analysis indicated an inverse association between 25(OH)D concentrations and the prevalence of GDM, others have not shown such an association [21, 22, 34–36]. In two cross-sectional studies conducted in mothers with sufficient levels of serum 25(OH)D [25(OH)D ≥ 50 nmol/L] or with a low risk for GDM, no significant difference in pregnancy 25(OH)D levels was observed between the GDM and control group [21, 34]. In a cohort of 1953 pregnant women with a mean serum 25(OH)D of 27.03 ng/ml, a higher serum level of 25(OH)D during 16-20-week gestation was associated with a higher prevalence of GDM (OR: 1.02; 95% CI: 1.00, 1.03) [35]. It is noteworthy that these studies predominantly involved subjects with sufficient vitamin D levels, emphasizing the need for high-quality cohort studies in participants with suboptimal vitamin D concentrations.

The genetic variations of VDR expression may influence the activity of vitamin D and the effect of supplementation in diabetic patients [37, 38]. In our study, *VDR* rs731236-G variant was associated with increased levels of 25(OH)D_3_, whereas *VDR* rs7975232-A was inversely associated with 25(OH)D_3_ levels. Similarly to our study, some evidence indicated that the SNPs of rs7975232 [39, 40] was associated with VD level, but another research showed no association [41, 42]. The conflict may be influenced by ethnicity, genotype frequencies, measurement methods of 25(OH)D_3_, or sample size in different studies. The compound of *VDR* and 25(OH)D_3_ might act on specific DNA sequences to regulate the activity of 25(OH)D_3_ [40]. The variants of rs7975232 might have a documented effect on the *VDR* protein level, and influence the activity of vitamin D in GDM patients.

The current study, so far as we know, is the first to examine the mediating role of 25(OH)D_3_ concentration in relationships of *VDR* SNPs and risk of GDM. It should be noted that the observed associations of *VDR* rs731236 and rs7975232 polymorphisms with GDM risk were directionally opposite. The rs731236 and rs7975232 are located at the three-primer untranslated region (3′-UTR) of the *VDR* gene, a region less likely to affect the amino acid sequence and the function of the gene. Based on these data, it was speculated that the rs731236 and rs7975232 variants might regulate *VDR* gene expression via mRNA binding, thereby affecting vitamin D efficacy, β-cell function and inflammatory responses, all of which affect diabetes progression [43–46]. The association of the *VDR* rs731236 and rs7975232 variants with 25(OH)D_3_ concentration may be a marker of the (unclear) effects of the variants that modify GDM risk. The present study lack of significant interactions between vitamin D level and VDR variants supports the idea that 25(OH)D_3_ concentrations acts primarily as a mediator rather than a potent effect modifier when considering vitamin D metabolic pathways involved in GDM, and the results are consistent with a previous study on GDM [47]. These findings highlight *VDR* variants might be more important than 25(OH)D_3_ concentrations in defining management strategies of vitamin D in GDM pregnancy.

Several limitations should be acknowledged in explaining the results. First, because of the season at sampling, latitude, and skin pigmentation considered as the most important influence factors of vitamin D requirements [48], participants in the current study were recruited at the same hospital, and assigned randomly to case and control groups matched by sampling season to minimize the influence of vitamin D requirements. Secondly, measurements of 25(OH)D_3_ were used by ELISA, which was a convenient and inexpensive method. Although the LC-MS/MS has advantages of low batch-to-batch variation and limit of detection [49, 50], previous studies demonstrated good overall agreement of measurement of free 25(OH)D_3_ between ELISA and LC-MS/MS [51]. In addition, we measured all samples in duplicate, and calculated the average of the two readings as the final value. Therefore, measuring the serum 25(OH)D_3_ concentration by ELISA still provides a reliable result for this study. Third, measurements of maternal 25(OH)D_3_ concentrations were only measured in the second trimester. However, the results from the Alberta Pregnancy Outcomes and Nutrition study showed no significant difference for maternal 25(OH)D_3_ level between the first and second trimesters (92.2 ± 25.6 nmol/L vs. 91.9 ± 25.0 nmol/L, *P* = 0.52) [52], indicating that measurement of maternal 25(OH)D_3_ concentrations in the second trimester was reasonable in the current study. Fourth, given the observational nature of the present study, residual confounding cannot be entirely ruled out, despite adjustment for a wide range of potential confounders. Fifth, the lack of information on physical activity and work type (e.g., shift work and occupation type) is a significant limitation of the study, especially given the potential influence of sedentary behaviors and evening shift work on developing GDM [53, 54]. Additionally, although the post-hoc power calculation using a two-sided alpha level of 0.05 indicated that the current sample size yielded sufficient power (> 89%) to detect the main associations, results of the study should be interpreted with caution due to limited sample size and the relatively wide CI for the mediation proportion of 25(OH)D_3_. Further prospective studies with larger sample sizes are required to clarify these statistically significant differences. Finally, since our participant were recruited from Henan province of China, the generalizability of results to other localities may be limited.

## Conclusions

In conclusion, *VDR* rs7975232 and rs731236 polymorphisms were associated with the risk of developing GDM. Higher 25(OH)D_3_ levels were associated with a decreased risk of GDM. Circulating 25(OH)D_3_ may partially mediate the association of *VDR* rs731236 and rs7975232 polymorphisms with GDM risk. Although the current study supports the vital role of the vitamin D metabolic pathway in GDM etiology, further cohort studies with larger sample sizes are warranted to replicate and extend these findings. 

## Supplementary Information

Below is the link to the electronic supplementary material.

Supplementary Material 1

Supplementary Material 2

## Data Availability

The data are not publicly available due to participants’ private information. However, data could be available by reasonable from the first author via email.

## Abbreviations

BMI: Body mass index
CIs: Confidence intervals
ELISA: Enzyme-linked immunosorbent assay
FPG: Fasting plasma glucose
GDM: Gestational diabetes mellitus
HWE: Hardy-Weinberg equilibrium
IADPSG: International Association of Diabetes and Pregnancy Study Groups
LD: Linkage disequilibrium
MAF: Minor allele frequency
NCBI: National Center for Biotechnology Information
OD: Optical density
OGTT: Oral glucose tolerance test
ORs: Odds ratios
PCR: Polymerase chain reaction
RCS: Restricted cubic splines
SD: Standard deviation
SNP: Single nucleotide polymorphism
VDR: Vitamin D receptor
1,25(OH)2D: 1,25-dihydroxyvitamin D
1-h PG: 1-h plasma glucose
2-h PG: 2-h plasma glucose
25(OH)D_3_: 25-hydroxyvitamin D_3_
