# Supplementary material for: Associations between genetic variants of vitamin D metabolic pathway and gestational diabetes mellitus: the potential mediation role of serum 25(OH)D3
Source: BMC Endocr Disord. 2026 Feb 24;26:91. doi: 10.1186/s12902-026-02199-w (PMC13041490; doi:10.1186/s12902-026-02199-w)
Supplement: Supplementary file 1 — Supplementary Material 1 [file 12902_2026_2199_MOESM1_ESM.docx]

**Pregnant Women's Health Survey Questionnaire**

Dear expectant mother,

Hello! Thank you for participating in the Gestational Diabetes Precision Nutrition Management Project. In the next 20 minutes, please cooperate with us to complete this survey questionnaire. The questionnaire will help us record the basic information of you and your family, as well as your lifestyle and health status before and during pregnancy. Please answer the questions based on your actual situation. There are no right or wrong answers to all the questions.

Thank you for your support!

Survey Date: [**] Year [**] Month [____] Day

Survey Gestational Age: [**] Weeks + [**] Days

Last Menstrual Period: [**] Year [**] Month [____] Day

Pregnant Woman's Name:

Medical Record Number:

Registration Number:

**Section 1: Basic Information**

1.Your phone number: [**]-[**]-[**] Hometown: [**] Province [____] City (County)

Permanent Address: [**] Province [**] City [**] County (District) [**] Street (Town)

2.Spouse's mobile phone number: [**]-[**]-[**] Hometown: [**] Province [____] City

3.Your ID number: [**]-[**]-[**]-[**]

4.Please fill in the birth years and months of you and your child's father.

Your birth year and month: [**] Year [**] Month; Spouse's birth year and month: [**] Year [**] Month

5.Please fill in the ethnic groups of you and your child's father.

Your ethnicity ( ) Spouse's ethnicity ( )

① Han

② Other ethnic groups

7.Please fill in your family situation.

7.1 Your marital status? ① Unmarried ② Married ③ Divorced ④ Other

7.2 Are you married to a close relative within three generations? ① No ② Yes, relationship

Section 2: Obstetric History

1.Menstrual history

1.1 How old were you when you had your first menstrual period? [____] years old

1.2 Is your menstrual cycle regular?

① Menstrual cycle fluctuates by no more than 7 days ② Menstrual cycle fluctuates by 7-10 days ③ Menstrual cycle shortens by more than 10 days ④ Menstrual cycle extends by more than 10 days

1.3 What is the average interval between your menstrual periods? [] days, and the average duration of each menstrual period is [] days.

1.4 In the past year, have you experienced "dysmenorrhea" during menstruation? ① No (skip to 1.5) ② Yes

1.4.1 Degree of dysmenorrhea: ① Mild (no treatment needed) ② Moderate (requires medication) ③ Severe (requires leave/bed rest)

1.5 In the year before this pregnancy, did you have amenorrhea for three consecutive months or longer?

① No ② Yes, [] times, the most recent amenorrhea lasted [] months ③ Unclear

Cause of amenorrhea:

2.Obstetric history

2.1 Have you been pregnant before? (Note: This does not include the current pregnancy)

① No (skip to Section 4) ② Yes, this is the [] time pregnant, and I have given birth to [] children

2.2 Please fill in the relevant details of any previous abnormal pregnancies.

① Natural miscarriage [] times ② Medical abortion/induced abortion [] times ③ Induced abortion [] times ④ Stillbirth [] times ⑤ Ectopic pregnancy [] times ⑥ Hydatidiform mole [] times ⑦ Defective fetus [____] times, name of defect: ________________________ ⑧ No abnormal pregnancies

2.3 Did you have any pregnancy complications or comorbidities in previous pregnancies? ① No (skip to Section 3) ② Yes

2.3.1 Specific names of pregnancy complications or comorbidities (multiple selections allowed)

① Pregnancy-induced hypertension ② Gestational diabetes ③ Thyroid disease ④ Maternal-fetal blood type incompatibility ⑤ Fetal growth restriction ⑥ Placental problems (A Placental abruption; B Placenta previa; C Placenta accreta) ⑦ Other

3.How long was the interval between your last miscarriage/delivery and this pregnancy? ① Less than half a year ② Half a year to 1 year ③ 1 year to 2 years ④ More than 2 years

4.Contraceptive use

4.1 Have you used long-term oral contraceptives or long-acting contraceptive injections for contraception? (e.g., Contraceptive Injection No. 1, Meieryi)

① No ② Yes, used for [____] months

4.2 Did you take emergency contraceptives after your last menstrual period? (e.g., Yuting, Anting, Huiting, Danmei, Kareding, etc.)

① No ② Yes

4.3 Was this pregnancy planned?

① No, this pregnancy was unintended (skip to Section 5) ② Yes, this pregnancy was planned

4.4 How many months did you prepare for this pregnancy without contraception?

① 1-3 months ② 4-6 months ③ 7-12 months ④ More than 1 year

5.Did you receive infertility treatment for this pregnancy? ① No (skip to Section 6) ② Yes

5.1 Methods of infertility treatment received (multiple selections allowed)

① Tubal surgery ② Treatment for endometriosis ③ Assisted reproductive technology ④ Western medicine treatment (hormones, contraceptives, etc.) ⑤ Traditional Chinese medicine treatment ⑥ Other

6.What was the method of conception for this pregnancy? ① Natural conception ② Artificial insemination ③ In vitro fertilization (please continue to answer 6.1)

6.1 If the method of conception for this pregnancy was "in vitro fertilization," please specify:

① In vitro fertilization and embryo transfer (IVF): A Fresh embryo B Frozen embryo C Unclear

② Intracytoplasmic sperm injection (ICSI): A Fresh B Frozen embryo C Unclear

③ Preimplantation genetic diagnosis (PGD): A Fresh embryo B Frozen embryo C Unclear

Section 3: Health Status

1.Have you undergone premarital or preconception check-ups?

① Only premarital check-up ② Only preconception check-up ③ Both premarital and preconception check-ups ④ Neither ⑨ Unclear

2.Height and weight

Your pre-pregnancy weight []. [] kg, current weight []. [] kg, height []. [] cm. Your spouse's weight []. [] kg, height []. [] cm.

1. Please fill in the birth conditions of you and your child's father:

|  | **Yourself** | **Your spouse** |
| --- | --- | --- |
| **Premature birth?** | **①No ②Yes ③Unknown** | **①No ②Yes ③Unknown** |
| Birth weight? | ①<2500 grams (<5 pounds) | ①<2500 grams (<5 pounds) |
|  | ②2500-4000 grams (5-8 pounds) | ②2500-4000 grams (5-8 pounds) |
|  | ③>4000 grams (>8 pounds) | ③>4000 grams (>8 pounds) |
|  | ④Unknown | ④Unknown |
| Was it a twin or multiple pregnancy? | ①Singleton ②Twin | ①Singleton ②Twin |
|  | ③Triplet or more | ③Triplet or more |

1. In the six months before pregnancy to the present, have you suffered from any chronic diseases (disease duration exceeding 14 days)? Diseases can be selected multiple times. Please fill in the onset date of each disease in turn, and in the "Current disease status" column, indicate the status of different diseases with the disease number. For example, if you have both respiratory system diseases such as ② asthma and ③ tuberculosis, and tuberculosis has been cured while asthma is still ongoing, the "Current disease status" should be filled in as "① Not cured ②; ③ Cured ③; [date of cure] (this date is for illustration only, please fill in the actual date of cure)."

| **Category** | **Disease Name** | **Taking Medication** | **Onset Date** | **Current Status of Disease** |
| --- | --- | --- | --- | --- |
| 4.1 Respiratory System Disease:① No ② Yes | ① Pharyngitis/Rhinitis  ② Asthma ③ Tuberculosis ④ Other: | ① No  ② Yes | □□Year□□Month□□ Day  □□Year□□Month□□ Day  □□Year□□Month□□ Day | ①Not cured ②Improved ③Cured ，□□Year□□Month□□ Day  ④Other： |
| 4.2 Digestive System  ① No ② Yes | ① Gastroenteritis ② Gastrointestinal bleeding/ulcer  ③ Hepatitis B  ④ Other: | ① No  ② Yes | □□Year□□Month□□ Day  □□Year□□Month□□ Day  □□Year□□Month□□ Day | ①Not cured ②Improved ③Cured ，□□Year□□Month□□ Day  ④Other： |
| 4.3 Cardiovascular and Hematological Diseases：  ① No ② Yes | ①Congenital heart disease ② Myocarditis ③Hypertension ④Anemia ⑤Other: | ① No  ② Yes | □□Year□□Month□□ Day  □□Year□□Month□□ Day  □□Year□□Month□□ Day | ①Not cured ②Improved ③Cured ，□□Year□□Month□□ Day  ④Other： |
| 4.4 Urogenital andReproductive System Diseases：  ① No ② Yes | ① Nephritis ② Cervicitis ③Vaginitis  ④ Other: | ① No  ② Yes | □□Year□□Month□□ Day  □□Year□□Month□□ Day  □□Year□□Month□□ Day | ①Not cured ②Improved ③Cured ，□□Year□□Month□□ Day  ④Other： |
| **Category** | **Disease Name** | **Taking Medication** | **Onset Date** | **Current Status of Disease** |
| 4.5 Neurological and Psychiatric Diseases：  ① No ② Yes | ① Epilepsy ② Depression  ③ Other: | ① No  ② Yes | □□Year□□Month□□ Day  □□Year□□Month□□ Day  □□Year□□Month□□ Day | ①Not cured ②Improved ③Cured ，□□Year□□Month□□ Day  ④Other： |
| 4.6 Endocrine and Metabolic Diseases:  ① No ② Yes | ① Hypothyroidism ② Hyperthyroidism ③Diabetes ④ Gout⑤ Phenylketonuria  ⑥Other: | ① No  ② Yes | □□Year□□Month□□ Day  □□Year□□Month□□ Day  □□Year□□Month□□ Day | ①Not cured ②Improved ③Cured ，□□Year□□Month□□ Day  ④Other： |
| 4.7 Connective Tissue and Rheumatic Immune Diseases：  ①否 ②是 | ①Systemic Lupus Erythematosus ②Arthritis ③Rheumatoid Arthritis ④Other: | ① No  ② Yes | □□Year□□Month□□ Day  □□Year□□Month□□ Day  □□Year□□Month□□ Day | ①Not cured ②Improved ③Cured ，□□Year□□Month□□ Day  ④Other： |
| 4.8 Other Diseases： ①否 ②是 | ①Severe Acne ②Toxoplasmosis ③Other： | ① No  ② Yes | □□Year□□Month□□ Day  □□Year□□Month□□ Day  □□Year□□Month□□ Day | ①Not cured ②Improved ③Cured ，□□Year□□Month□□ Day  ④Other： |

5.Did the child's father have any of the above chronic diseases within 3 months before your pregnancy? ①No (skip to 6) ②Yes

5.1 If yes, the specific disease name is _________

5.3 Date of onset: □□□□Year □□Month □□Day or ⑨Unclear

5.3 Current status of the disease ①Not Cured ②Improved ③Cured, □□□□Year □□Month □□Day ④Other: _________

6.Are you and the child's father allergic to the following substances? (Multiple choices allowed)

| Yourself (Please check) | Child's Father (Please check) |
| --- | --- |
| ①None (skip to 10) | ①None (skip to 10) |
| ②Food: ____________________ | ②Food: ____________________ |
| ③Medicine: ____________________ | ③Medicine: ____________________ |
| ④Pollen | ④Pollen |
| ⑤Dust | ⑤Dust |
| ⑥Animal Dander | ⑥Animal Dander |
| ⑦Other: ____________________ | ⑦Other: ____________________ |

7.Within your family's direct relatives over three generations, are there any congenital/hereditary diseases? (Multiple choices allowed)

①No ②Yes, the relationship to you is ________, disease name ________ (fill in the options below)

8.Within the child's father's family's direct relatives over three generations, are there any congenital/hereditary diseases? (Multiple choices allowed)

Section 4: Current Pregnancy

1.Have you experienced threatened miscarriage since pregnancy? ① No (skip to Question 2) ② Yes

1.1 How many times have you had threatened miscarriage: [____] times

1.2 How long did the longest episode last: [____] days

1.3 When did the longest episode occur: at [____] weeks of pregnancy

1.4 What were the symptoms of the longest episode: ① Vaginal bleeding ② Abdominal pain ③ Back pain

1.5 How much bleeding was there during the most severe episode: ① A lot ② Much ③ Moderate ④ Little ⑤ Very little

1.6 After the symptoms of threatened miscarriage, did you receive treatment? (1) No (2) Yes: ① Western medicine ② Traditional Chinese medicine ③ Bed rest

2.Have you had a cold since pregnancy? ① No (skip to Question 3) ② Yes

2.1 Did you take any medication after having a cold? ① No ② Yes (please fill in the medication details in Question 9)

3.Have you had a fever (temperature ≥37.3⁰C) since pregnancy? ① No (skip to Question 4) ② Yes, for [____] days

3.1 Did you take any medication after having a fever? ① No ② Yes (please fill in the medication details in Question 9)

4.Have you been diagnosed with pregnancy-induced hypertension (systolic pressure ≥140 or diastolic pressure ≥90 mmHg or both)? ① No (skip to Question 5) ② Yes (diagnosed at [____] weeks of pregnancy)

4.1 Are you taking any medication: ① No ② Yes (please fill in the medication details in Question 9)

4.2 Is blood pressure control satisfactory: ① Unsatisfactory ② Satisfactory (blood pressure <140/90 mmHg

5.Have you been diagnosed with gestational diabetes: ① No (skip to Question 6) ② Yes

5.1 Methods of treatment for diabetes or gestational diabetes: ① Diet control ② Exercise ③ Oral hypoglycemic drugs ④ Insulin

5.2 Is blood glucose control satisfactory: ① No ② Yes ⑨ Unclear

6.Have you suffered from anemia during this pregnancy? ① No (skip to Question 7) ② Yes ⑨ Unclear

6.1 Severity of anemia: ① Mild anemia ② Moderate anemia ③ Severe anemia ⑨ Unclear

7.Have you developed intrahepatic cholestasis of pregnancy: ① No (skip to Question 8)② Yes (diagnosed at [____] weeks of pregnancy)

7.1 Have you experienced skin itching: ① No ② Yes

7.2 What medications have you taken? ① No medication ② Medication taken (please fill in the medication details in Question 9)

8.Since pregnancy, apart from the diseases mentioned above, have you been diagnosed with any of the following diseases by a doctor?

① No (skip to Question 9) ② Yes, specifically:

| **Disease Name** | **Occurrence** | **Onset Time** | **Duration** | **Hospitalization** |
| --- | --- | --- | --- | --- |
| Nausea/Vomiting | ①NO ②Yes | □□weeks | □□days | ①NO ②Yes |
| Dyspepsia | ①NO ②Yes | □□weeks | □□days | ①NO ②Yes |
| Heartburn | ①NO ②Yes | □□weeks | □□days | ①NO ②Yes |
| Constipation | ①NO ②Yes | □□weeks | □□days | ①NO ②Yes |
| Leg cramps | ①NO ②Yes | □□weeks | □□days | ①NO ②Yes |
| Periodontal disease | ①NO ②Yes | □□weeks | □□days | ①NO ②Yes |
| Oral mucosal disease | ①NO ②Yes | □□weeks | □□days | ①NO ②Yes |
| Cavities | ①NO ②Yes | □□weeks | □□days | ①NO ②Yes |
| Hemorrhoids | ①NO ②Yes | □□weeks | □□days | ①NO ②Yes |
| Pelvic inflammatory disease | ①NO ②Yes | □□weeks | □□days | ①NO ②Yes |
| Urinary tract infection | ①NO ②Yes | □□weeks | □□days | ①NO ②Yes |
| Genital herpes | ①NO ②Yes | □□weeks | □□days | ①NO ②Yes |
| Reproductive tract inflammation | ①NO ②Yes | □□weeks | □□days | ①NO ②Yes |
| Heart disease | ①NO ②Yes | □□weeks | □□days | ①NO ②Yes |
| Varicose veins in the lower limbs | ①NO ②Yes | □□weeks | □□days | ①NO ②Yes |
| Hypothyroidism | ①NO ②Yes | □□weeks | □□days | ①NO ②Yes |
| Hyperthyroidism | ①NO ②Yes | □□weeks | □□days | ①NO ②Yes |
| Epilepsy | ①NO ②Yes | □□weeks | □□days | ①NO ②Yes |
| Depression | ①NO ②Yes | □□weeks | □□days | ①NO ②Yes |
| Other 1 | ①NO ②Yes | □□weeks | □□days | ①NO ②Yes |
| Other 2 | ①NO ②Yes | □□weeks | □□days | ①NO ②Yes |
| Other 3 | ①NO ②Yes | □□weeks | □□days | ①NO ②Yes |

9.Since pregnancy, please list all medications used, including frequency and duration of use. (Excluding trace element preparations, vitamins, and other nutritional supplements)

① No medications taken (skip to Question 10) ② Medications taken (please fill in the table below)

| Medication Name | Formulation* | Method of Use* | Onset Time | Stopping  Time | Actual Use Days of Use | Frequency of Use | Single dose |
| --- | --- | --- | --- | --- | --- | --- | --- |
| 1 |  |  |  |  |  |  |  |
| 2 |  |  |  |  |  |  |  |
| 3 |  |  |  |  |  |  |  |
| 4 |  |  |  |  |  |  |  |
| 5 |  |  |  |  |  |  |  |
| 6 |  |  |  |  |  |  |  |

Formulation: ① Liquid ② Gas (aerosol, spray) ③ Solid ④ Semisolid (ointment, suppository, paste)

Method of Use: ① Oral ② Intramuscular injection ③ Intravenous injection ④ Respiratory ⑤ Transdermal ⑥ Sublingual ⑦ Intravaginal ⑧ Other

10.Since pregnancy, have your allergies to certain substances changed? ① No

② Yes, no longer allergic to ③ Yes, started being allergic to

Section 7: Health Behaviors

1.Have you ever smoked? (Even just one cigarette counts) ① No (skip to Question 3) ② Yes

2.Please fill in your smoking habits for different periods:

| Smoke | Before Pregnancy | | During pregnancy |
| --- | --- | --- | --- |
|  | 1 year before pregnancy ~3 months before pregnancy | 3 months before pregnancy |  |
| Frequency | ① Never smoked ② Only smoked [] cigarettes ③ Average [] cigarettes/[] days, smoked for []. [____] years | ①Never smoked ②Only smoked [] cigarettes | ①Never smoked ②Only smoked [] cigarettes |
|  |  | ③Average [] cigarettes/[] days | ③Average [] cigarettes/[] days |

3.Did your spouse smoke before your pregnancy? ① No ② Yes, average smoking amount is: ① <5 cigarettes/day ② 5-9 cigarettes/day ③ 10-19 cigarettes/day ④ 20-29 cigarettes/day ⑤ 30-39 cigarettes/day ⑥ ≥40 cigarettes/day

4. Please fill in your exposure to second-hand smoke for different periods:

| Question | Before Pregnancy | | During pregnancy |
| --- | --- | --- | --- |
|  | 1 year before pregnancy - 3 months before pregnancy | 3 months before pregnancy |  |
| 4.1 Are there smokers living with you? | ① No ② Yes, [] people  ③ Don't remember | ① No ② Yes, [] people  ③ Don't remember | ① No ② Yes, [] people  ③ Don't remember |
| 4.2 Do you smell smoke at home? | ① Never ② Yes, [] years | ① Never ② Yes, [] days/week | ① Never ② Yes, [] days/week |
| 4.3 Do you smell smoke at work? | ① Never ② Yes, []years ③ No work | ① Never ② Yes, [] days/week ③ No work | ① Never ② Yes, [] days/week ③ No work |

5.1 How often did you drink alcohol in the three months before pregnancy?

① Never ② <1 time/month ③ 1-3 times/month ④ 1-3 times/week ⑤ 4-6 times/week ⑥ ≥7 times/week

5.2 How often have you drunk alcohol in the three months after pregnancy?

① Never ② <1 time/month ③ 1-3 times/month ④ 1-3 times/week ⑤ 4-6 times/week ⑥ ≥7 times/week

6. Does your spouse usually drink alcohol?

① Never ② <1 time/month ③ 1-3 times/month ④ 1-3 times/week ⑤ 4-6 times/week ⑥ ≥7 times/week

The questionnaire ends here. Thank you very much for your cooperation. Wishing you all the best!
