# Supplementary material for: Associations between genetic variants of vitamin D metabolic pathway and gestational diabetes mellitus: the potential mediation role of serum 25(OH)D3
Source: BMC Endocr Disord. 2026 Feb 24;26:91. doi: 10.1186/s12902-026-02199-w (PMC13041490; doi:10.1186/s12902-026-02199-w)
Supplement: Supplementary file 2 — Supplementary Material 2 [file 12902_2026_2199_MOESM2_ESM.docx]

**Supplementary Materials**


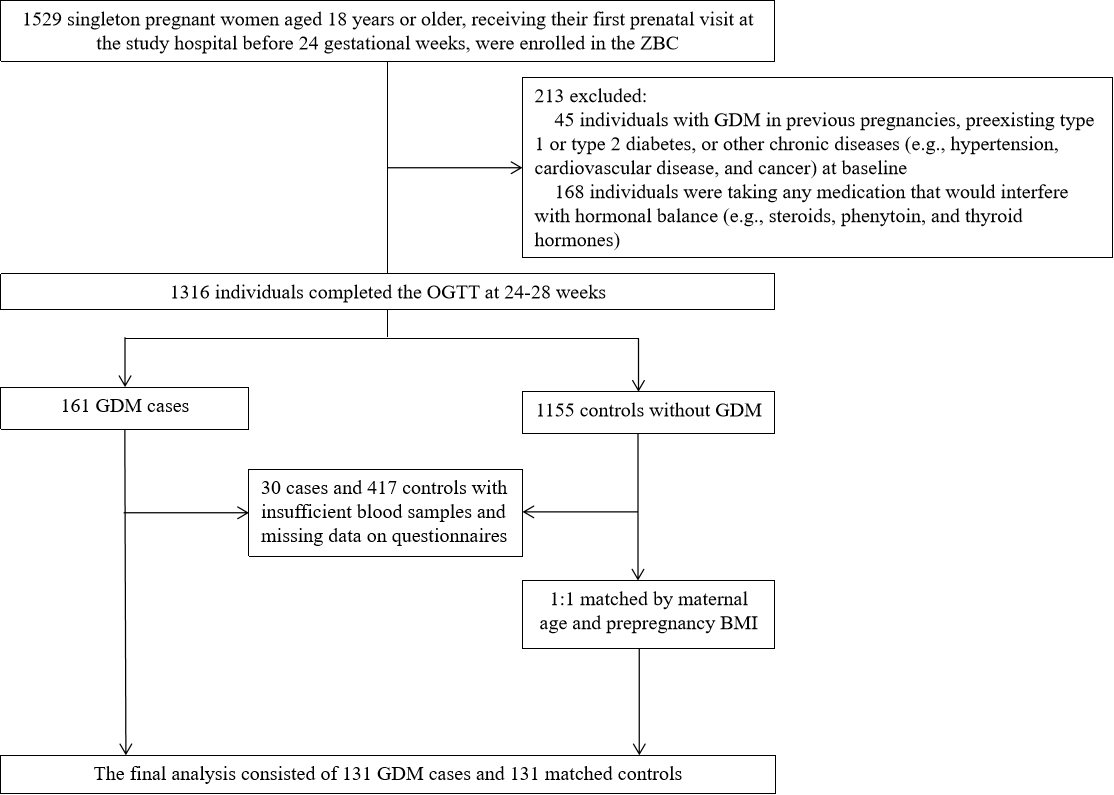


**Figure S1.** Flowchart of study participants in the nested case-control study.

Abbreviations: BMI: body mass index; GDM, gestational diabetes mellitus; OGTT, 75-g oral glucose tolerance test; ZBC, the Zhengzhou Birth Cohort.

**Table S1.** Single nucleotide polymorphism and test of Hardy–Weinberg equilibrium

| Gene | SNP | location | allele | Homozygote (minor  allele) | Heterozygote | Homozygote (alternative allele) | HEW(χ^2^) | HWE(*P*) |
| --- | --- | --- | --- | --- | --- | --- | --- | --- |
| *VDR* | rs731236 | 12q13.11 | A/G | 3 (1.15) | 52 (19.85) | 207 (79.00) | 0.26 | 0.88 |
|  | rs7975232 | 12q13.11 | C/A | 43 (16.67) | 102 (39.53) | 113 (43.80) | 0.01 | 0.99 |
|  | rs739837 | 12q13.11 | G/T | 25 (9.54) | 98 (37.41) | 139 (53.05) | 0.06 | 0.97 |
|  | rs1544410 | 12q13.11 | C/T | 2 (0.78) | 39 (14.89) | 219 (84.23) | 0.00 | 1.00 |
|  | rs2228570 | 12q13.11 | G/A | 47 (18.08) | 129 (49.62) | 84 (32.30) | 0.26 | 0.88 |
| *CYP2R1* | rs10741657 | 11p15.2 | G/A | 50 (19.53) | 126 (49.22) | 80 (31.25) | 1.84 | 0.40 |
|  | rs12794714 | 11p15.2 | G/A | 36 (14.29) | 121 (48.01) | 95 (37.70) | 1.85 | 0.40 |
| *CYP24A1* | rs2248359 | 20q13.2 | C/T | 32 (12.22) | 121 (46.18) | 109 (41.60) | 1.53 | 0.47 |
| *CYP27B1* | rs10877012 | 12q14.1 | T/G | 39 (14.89) | 137 (52.29) | 86 (32.82) | 1.00 | 0.61 |

Abbreviations: *VDR*, vitamin D receptor.

**
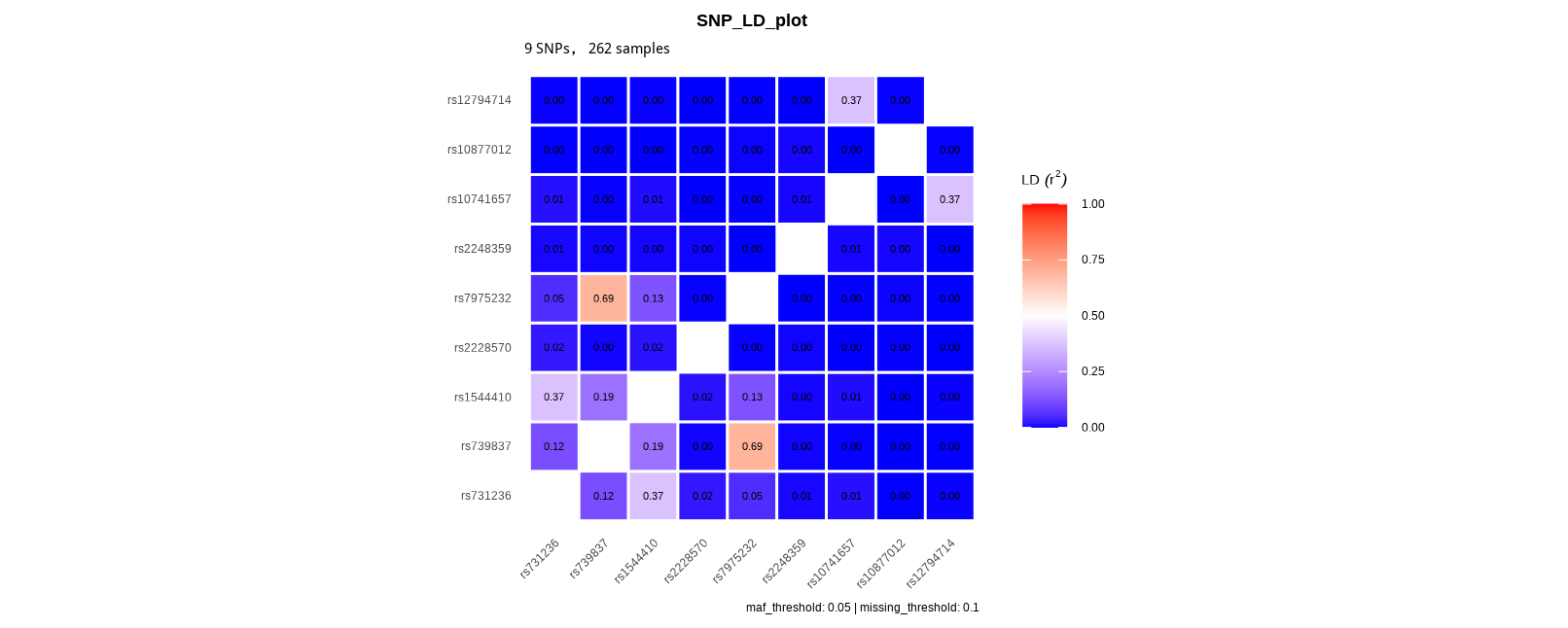
**

**Figure S2.** The linkage disequilibrium (LD) plot of SNPs.

**Table S2.** Mediation analysis of serum 25(OH)D_3_ on associations between SNPs and GDM risk

| SNP | Total effect | | Indirect effect | | Direct effect | | Proportion mediated, % (95%CI) | *P* value |
| --- | --- | --- | --- | --- | --- | --- | --- | --- |
|  | Coefficients (95% CI) | *P* value | Coefficients (95% CI) | *P* value | Coefficients (95% CI) | *P* value |  |  |
| rs731236 | -0.24 (-0.36, -0.12) | <0.01 | -0.06 (-0.12, -0.01) | 0.02 | -0.18 (-0.29, -0.04) | <0.01 | 24.7% (2.8%, 76.0%) | 0.02 |
| rs7975232 | 0.25 (0.11, 0.39) | <0.01 | 0.07 (0.03, 0.13) | <0.01 | 0.17 (0.05, 0.33) | 0.04 | 29.6% (11.4%, 70.0%) | <0.01 |

Abbreviations: BMI, body mass index; CI, confidence interval; GDM, gestational diabetes mellitus; SNPs, single nucleotide polymorphisms; 25(OH)D_3_, 25-hydroxyvitamin D3. The mediation analyses were adjusted for age, pre-pregnancy BMI, previous poor pregnancy outcome history.
